# Which factors influence plan reuse in a sequential posture selection task?

**DOI:** 10.3389/fpsyg.2025.1423408

**Published:** 2025-02-25

**Authors:** Christoph Schütz

**Affiliations:** ^1^Faculty of Psychology and Sports Science, Bielefeld University, Bielefeld, Germany; ^2^Center for Cognitive Interaction Technology, Bielefeld University, Bielefeld, Germany

**Keywords:** hysteresis, motor plan reuse, posture selection, influencing factors, sequential effects

## Abstract

In a sequential posture selection task, we reuse former motor plans to reduce cognitive planning cost. The resulting persistence in the former posture, termed motor hysteresis, can serve as a proxy for the percentage of motor plan reuse (PoR). A recent study showed a significant drop in PoR if participants were asked to skip every second drawer in a sequential drawer opening task. In the current study, we sought to disentangle four confounded factors that were potentially responsible for this drop in PoR: a change of (1) spatial distance, (2) digit distance, (3) number of drawers, or (4) context (presence of skipped drawers). To this end, two groups of participants were tested in a series of sequential drawer tasks, where each of the four potential influencing factors was varied independently. PoR was calculated as the dependent variable. Participants displayed a hysteresis effect in all ordered tasks, but the PoR was only reduced by an increase in spatial distance. The three remaining factors had no significant effect. This finding indicates that motor planning is only affected by local (spatial) parameters of the task, but not by context factors (digits, skipped drawers) or global parameters such as the number of drawers.

## 1 Introduction

In a predictable, sequential motor task, former motor plans are reused and modified for subsequent movements. This results in a persistence in the former posture, termed *motor hysteresis* (Kelso et al., 1994). For example, if participants open a column of drawers in a descending sequence, they start in a pronated posture at the highest drawer and persist in a more pronated posture throughout the rest of the sequence. In an ascending sequence, in contrast, they start and subsequently persist in a more supinated posture (Schütz and Schack, 2013; Schütz et al., 2011). Such hysteresis in posture selection has been reliably demonstrated in a number of studies (Schütz et al., 2016; Schütz and Schack, 2020, 2019b, 2022).

According to the *plan-modification hypothesis*, the reuse of former motor plans reduces the cognitive cost of motor planning (Rosenbaum et al., 2007). The creation of a reaching movement plan involves a series of sensorimotor transformations to translate the retinal image into a muscle activation pattern that guides the hand to a target position (Jordan and Wolpert, 1999). Due to these transformations, the creation of a motor plan from scratch is associated with a cognitive cost. In a predictable, sequential motor task, people therefore do not create a new motor plan for each movement, but instead reuse and modify the former plan (Rosenbaum and Jorgensen, 1992).

While, in theory, motor hysteresis could also result from low pass properties of the muscular system, several studies indicate that hysteresis reflects a cognitive effect of motor planning. Van der Wel et al. (2007) asked participants to contact a series of targets on a tabletop in time with a metronome. If an obstacle had to be cleared between two targets, the jump peak height afterwards decreased only gradually. The hysteresis effect even persisted if participants cleared the obstacle with one hand and continued the progression with the other, thus discarding low pass properties of the muscle as the sole explanation for hysteresis.

Schütz and Schack (2013) asked participants to open a column of drawers in ordered sequences. If the mechanical cost of the task was temporarily increased by adding a counterforce to one of the drawers, the hysteresis effect was reduced. The reduction persisted after the mechanical cost was reset to its initial value, indicating that a lasting cognitive representation of the task properties had been formed. The *cost-optimization hypothesis* (Schütz et al., 2016) states that hysteresis effect size is a function of the cognitive cost of motor planning and the mechanical cost of motor execution, and that our motor system seeks to minimize the total cost of a movement. Thus, the optimal percentage of motor plan reuse (PoR) depends on the ratio of both cost factors.

Apart from the mechanical cost of the task, other factors have been found that affect the PoR: Jax and Rosenbaum (2007, 2009) asked participants to execute a center-out pointing task and to circumvent obstacles in some of the trials. In trials without an obstacle, hand paths remained more curved if an obstacle had recently been circumvented, indicating path planning hysteresis. The hysteresis effect depended on the time delay between subsequent trials and was almost completely eliminated if trials were delayed by more than 1,000 ms (Jax and Rosenbaum, 2009). Another potential factor tested by the authors was the predictability of the sequence: hand path curvature and, thus, path hysteresis, did not differ between predictable and unpredictable sequences of obstacles (Jax and Rosenbaum, 2007).

In contrast, hysteresis in posture selection is affected by the predictability of the sequence. Schütz et al. (2011) asked participants to execute both an unpredictable (randomized) and predictable (ordered) sequential drawer opening task. Hysteresis was present in the predictable condition but absent in the unpredictable one. In a more recent study, the authors applied a mathematical model that allowed to calculate the PoR in arbitrary sequences of drawers (Schütz and Schack, 2019a). In ordered sequences, participants on average reused 20.0% of the previous motor plan. In randomized sequences, average PoR dropped significantly (to 2.0%), indicating that hysteresis in a posture selection task was highly affected by the predictability of the task.

The mathematical model applied presumes a sigmoid optimal grasp posture function and a fixed PoR, since the cognitive and mechanical cost should be the same for all drawers (Schütz and Schack, 2019a). The model captured over 98.6% of the posture variance in all conditions tested, representing an almost perfect replication of participants’ behavioral data. The study, however, yielded one unexpected result: In addition to the randomized and ordered conditions, a modified ordered condition was examined in which participants were asked to skip every second drawer in the sequence. As both the cognitive and mechanical cost were the same as in the ordered condition, the authors assumed that the modified PoR would also be identical to the ordered PoR (15.6%). Instead, the PoR in the modified condition was considerably reduced (6.8%).

This reduction in hysteresis could not be attributed to any of the previously reported influencing factors (ratio of cognitive to mechanical cost, delay, predictability), as those were the same in both conditions. Therefore, it suggested the existence of another, as yet unknown, factor responsible for the reduction. Unfortunately, by switching from an ordered to a skipped ordered task, the authors changed multiple factors at once, thus creating a confound: (1) the spatial distance between the opened drawers (drawers were further apart), (2) the digit distance (digit sequence on the opened drawers became 1, 3, 5, … instead of 1, 2, 3, …), (3) the number of opened drawers (reduced from nine to five), and (4) the task context (participants were asked to skip all even drawers but they were still present in the shelf, which might have reduced the perceived binding between the opened odd drawers).

In the current study, we sought to resolve the confound by varying each of those factors in isolation. To this end, we tested participants in a series of sequential drawer opening tasks. We designed two experiments, one in which we changed the factors (1) spatial distance and (2) digit distance and one in which we changed the factors (3) number of drawers and (4) context. The PoR was calculated as the dependent variable by the mathematical model. Based on the results of the previous study (Schütz and Schack, 2019a), we expected at least one of the factors to have a significant effect on the PoR, that is, on the planning behavior of the participants in a sequential task, and thus to identify the yet unknown influencing factor(s).

## 2 Materials and methods

### 2.1 Power analysis

An *a priori* power analysis was calculated using the SPSS (28, IBM Corp., Armonk, NY) MANOVA procedure (D’Amico et al., 2001), which requires the number of participants for each group, means and standard deviations for the dependent variables, and correlations between the variables. Values were taken from the ordered pretest of the study by Schütz and Schack (2013), which was similar to the current experiment. Presuming a comparable effect size, 15 participants are sufficient to achieve a power of over 0.95 for detecting a main effect of the within subject factor ‘order’, indicating hysteresis.

### 2.2 Participants

In each experiment, 24 students [Experiment 1: 10 female, 14 male, age 24.6 ± 3.7 (SD) years; Experiment 2: 12 female, 12 male, age 24.0 ± 4.1 years] from Bielefeld University participated in exchange for course credit. In Experiment 1, 22 participants were right handed [handedness score (HS) 1.00 ± 0.00, one left handed (HS –0.60), and one ambidextrous (HS –0.14)] according to the revised Edinburgh inventory (Oldfield, 1971). In Experiment 2, 23 participants were right handed (HS 1.00 ± 0.00) and one left handed (HS –0.57). Participants reported no known neuromuscular disorders and were naïve to the purpose of the study. Each participant read a detailed set of instructions on the task and provided written informed consent before the experiment. The study was approved by the Bielefeld University ethics committee and conformed to the 1964 Declaration of Helsinki (World Medical Association, 2013).

### 2.3 Apparatus

**Figure 1:**
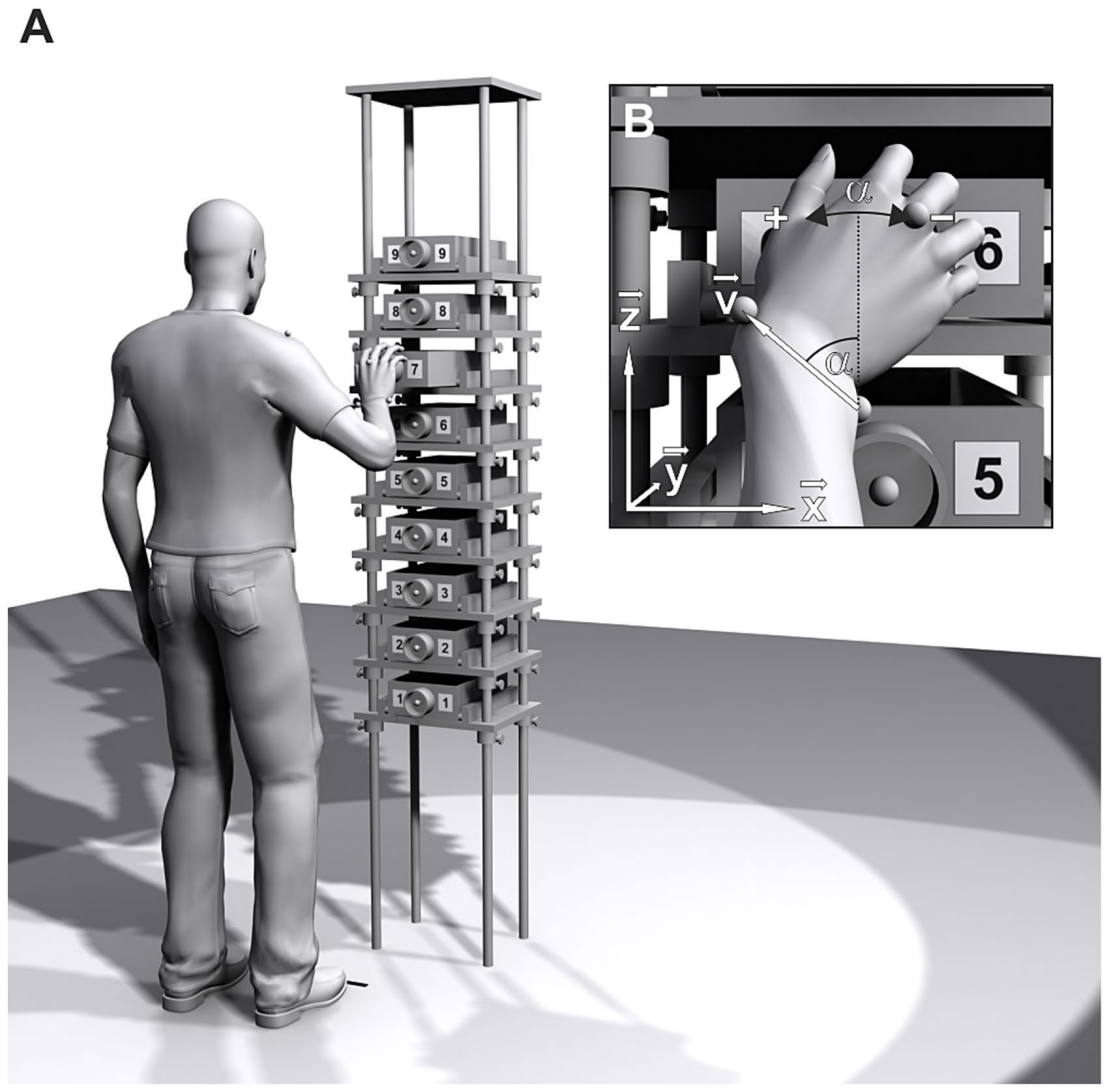
**(A**) Schematic of the experimental setup. Drawer height, spacing, and participant’s position are adjusted to the participant’s shoulder height and arm length. **(B)** Pro/supination angle *α*. The projection of the wrist vector **v** onto the drawer face (**x**-**z**-plane) at the moment of drawer grasp is used to calculate α.

The apparatus used was a tall metal frame (222 cm high, 40 cm wide and 30 cm deep) with nine wooden shelves (Figure 1A). A wooden drawer (8.5 cm high, 20 cm wide and 30 cm deep; pullout range 21.5 cm) was placed on each shelf. At the center of each drawer front, a gray plastic ring with a diameter of 7 cm and a depth of 4 cm was affixed. To the left and right of the knob, a number from 1 (lowest) to 9 (highest) was attached.

### 2.4 Preparation

Ten retro reflective markers were attached to the thorax and right arm of the participant (cf. Schütz and Schack, 2013 for a full list). In the current study, only four of those anatomical landmarks were used for the analysis: the most cranial point of the *acromion* (*AC*), the *radial* (*RS*) and *ulnar* (*US*) *styloid* process, and the back of the third *metacarpal* (*MC*). The approximate height of the shoulder joint center (0.97 × *height of AC*) and length of the arm (|*AC* – *RS|*) of the participant were measured in a T-pose (arms extended sideways and palms pointed forward).

The center of drawer #7 was aligned with the height of the shoulder joint center. Drawer spacing was set to 0.25 × *arm length*. The participant was positioned 1.00 × *arm length* in front of the setup and 0.33 × *arm length* to the left of the drawer centers. The participant’s position was marked by two strips of black tape: point of the toes and median plane of the body (Figure 1A).

### 2.5 Procedure

Each experiment consisted of six tasks. Each task contained up to eight sequences of trials. A single trial was defined as the opening and closing of one drawer. Each trial started from an initial position, with the palm of the hand touching the thigh. The participant had to (1) raise the arm to the drawer, (2) grasp the handle with a five-finger grip, (3) fully open the drawer, (4) close the drawer, and (5) return to the initial position. As hysteresis in the current posture selection task does not differ between the dominant and non-dominant hand (cf. Schütz and Schack, 2019b), all participants performed the task with their right hand, irrespective of handedness.

In Task 1, the participant performed four randomized sequences of all nine drawers (4 repetitions × 9 drawers; 36 trials). A list of pseudo-random (Mersenne twister algorithm; Matsumoto and Nishimura, 1998) permutations was created before the experiment. From the list, the experimenter announced the next drawer number as soon as the arm was back in the initial position.

**Figure 2:**
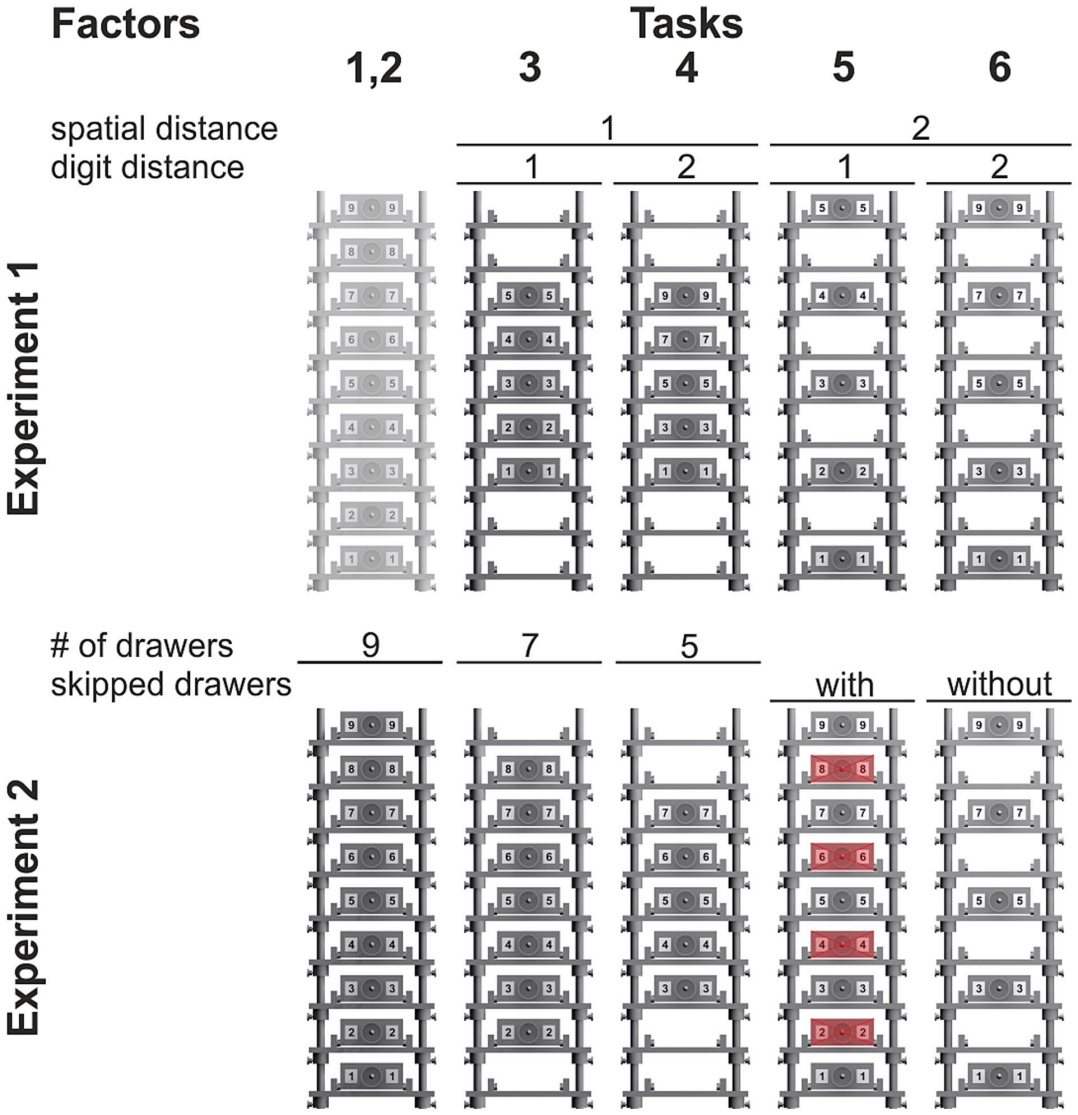
Schematics of the drawer setup for the individual sequential tasks. In Experiment 1 (*top*), Tasks 3 to 6, the factors ‘spatial distance’ and ‘digit distance’ are varied in a 2 × 2 design. In Experiment 2 (*bottom*), the factor ‘number of drawers’ is varied in Tasks 2 to 4 and the factor ‘context/skipped drawers’ is varied in Tasks 5 and 6.

In Tasks 2 to 6, the participant performed eight ordered sequences of trials each: four ascending, four descending. Sequence order was pseudo-randomized (see above) with no more than two repetitions of the same order in a row. The experimenter did not announce drawer numbers, but only the order of the sequence (‘from top to bottom’/‘from bottom to top’). The participant executed the trials of each sequence on his/her own. The drawer layout in Tasks 2 to 6 differed between experiments:

In Experiment 1, Task 2 consisted of all nine drawers (2 orders × 4 repetitions × 9 drawers; 72 trials; Figure 2, top). It was added to make the experiment more similar to Experiment 2, but was not used to analyze any factor’s influence on hysteresis. Tasks 3 to 6 consisted of only five drawers each and contained all possible combinations of spatial and digit distance (2 sequences × 4 repetitions × 5 drawers; 40 trials). In Tasks 3 and 4, drawers were placed on shelves 3, 4, 5, 6, and 7 and, thus, had a spatial distance of 1; in Tasks 5 and 6, drawers were placed on shelves 1, 3, 5, 7, and 9 and had a spatial distance of 2. In Tasks 3 and 5, drawers were labeled 1, 2, 3, 4, and 5 and, thus, had a digit distance of 1; in Tasks 4 and 6, drawers were labeled 1, 3, 5, 7, and 9 and had a digit distance of 2.

In Experiment 2, Tasks 2 to 4 consisted of nine, seven, and five drawers, respectively (2 orders × 4 repetitions × 9/7/5 drawers; 72/56/40 trials; Figure 2, bottom). Only the number of drawers was varied in these three tasks, spatial and digit distance were the same. In Task 3, drawers were placed on shelves 2–8 and labeled accordingly; in Task 4, drawers were placed on shelves 3–7. Tasks 5 and 6 consisted of five drawers only (2 sequences × 4 repetitions × 5 drawers; 40 trials). In Task 5, drawers were placed on all nine shelves, but participants were asked to skip every second drawer. Thus, a single sequence consisted only of the drawers 1, 3, 5, 7, and 9. In Task 6, drawers were only placed on shelves 1, 3, 5, 7, and 9 and labeled accordingly. Thus, spatial distance, drawer distance, and number of drawers were the same in both tasks, but the context varied.

Each participant conducted Task 1 first, to get accustomed to the experiment. Postures in randomized tasks are unaffected by hysteresis (Schütz et al., 2011; Schütz and Schack, 2022) and, thus, should not affect the subsequent tasks. The order of Tasks 2–6 was randomized across participants.

Before each task, the position of the participant in front of the apparatus was checked based on the floor marks. Participants had a resting period of 30 s between each sequence and of 2 min between each task. The entire experiment lasted approximately 60 min.

### 2.6 Kinematic analyses

Movement data were recorded by a Vicon MX (Vicon Motion Systems, Oxford, UK) motion capture system. Marker trajectories were reconstructed in Vicon Nexus 2.11, labeled manually, and exported to MATLAB (2021b, The MathWorks, Natick, MA) for data analysis. The laboratory’s coordinate system was defined with the **x**-axis pointing to the right, the **y**-axis pointing to the front and the **z**-axis pointing upwards while standing in front of the apparatus (Figure 1B).

To identify the moment of drawer grasp for each trial, the **y**-component (perpendicular to the drawer face, Figure 1B) of the *metacarpal* marker (*MC*) was analyzed. Its trajectory started from a low initial value (the initial posture) and exhibited two local maxima before returning to the initial value. The first local maximum, which corresponded to the moment of drawer grasp., was used to calculate the pro/supination angle *α*.

For the calculation of α, the wrist axis was projected onto the drawer face (**x**-**z**-plane, Figure 1B). A direction vector **v** was defined, pointing from *US* to *RS*: **v** = *RS* – *US*. From the vector components v_x_ and v_z_, the pro/supination angle α was calculated with the four-quadrant inverse tangent function of MATLAB.

**Figure 3:**
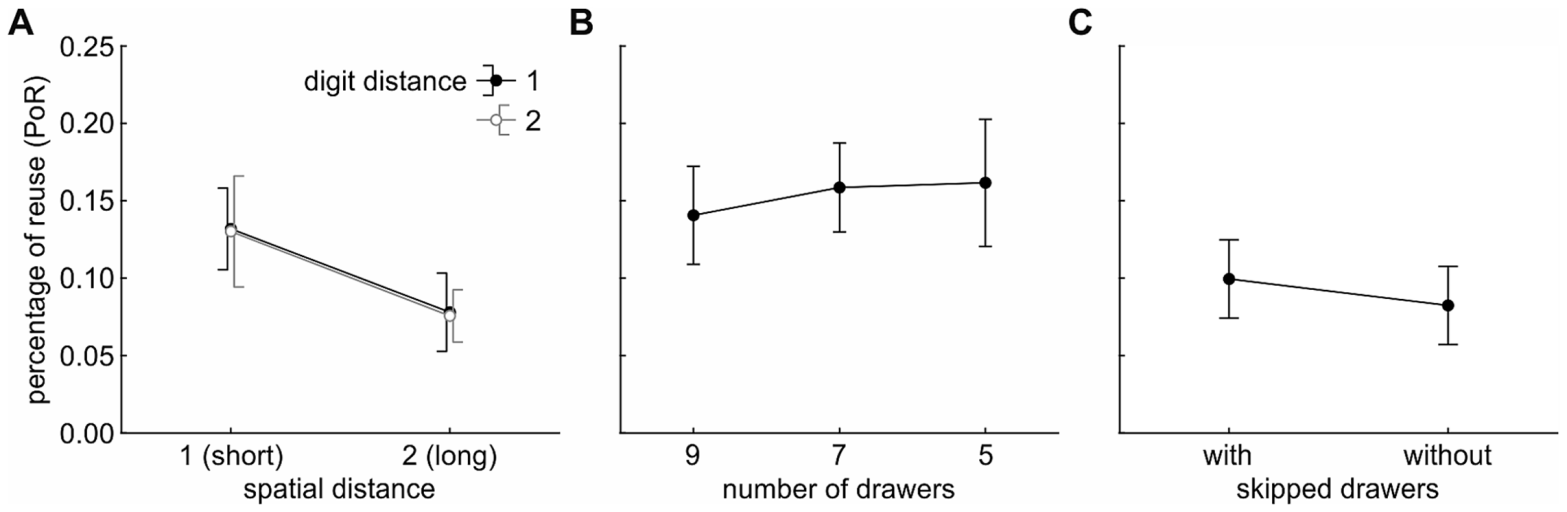
Percentages of reuse (PoR) split by condition. **(A)** PoR split by ‘spatial distance’ and ‘digit distance’. **(B)** PoR split by ‘number of drawers’. **(C)** PoR split by ‘context/skipped drawers’. *Data points* show the average across participants. *Error bars* indicate 95% (within-subject) confidence intervals.

The five-parameter model of Schütz and Schack (2019a) was applied to the pro/supination angle data of each participant and task. The model assumes that the optimal posture (i.e., pro/supination angle) at each drawer is a sigmoid function of drawer and that the hysteresis effect (i.e., difference between ascending and descending sequences) results from a partial reuse of the former posture. The posture at each drawer (except for the first drawer in the sequence) is calculated as a mixture of the optimal posture for that drawer and of the posture at the former drawer. The percentage of reuse (PoR) defines how many percent of the former posture are in the mixture (cf. Schütz and Schack, 2019a; Figure 3): With a PoR of 100%, the current posture is identical to the former posture; with a PoR of 0%, the current posture is identical to the optimal posture.

For the model to calculate the PoR in each condition, data from all trials of a single participant must be entered into the model, including the measured pro/supination angles, the corresponding drawer sequences (which define whether drawers were opened in random, ascending, or descending order), and a unique identifier for each task/experimental condition. The model parameters are then fitted to the measured data using least squares optimization (Marquardt, 1963; Levenberg, 1944). The four parameters for the sigmoid optimum curve are estimated across all tasks, while an individual fifth parameter for each condition (the PoR) is used to reproduce and quantify the hysteresis effect size.

In both experiments, the data from all six tasks was used to estimate the parameters of the sigmoid optimum curve. An individual fifth parameter (the PoR) was used for the Tasks 2 to 6, respectively, to calculate the percentage of motor plan reuse in each condition. The calculated PoR values were used as the dependent variable in all subsequent analyses. In Experiment 1, the PoR values of Tasks 3 to 6 were analyzed statistically; in Experiment 2, the PoR values of Tasks 2 to 6 were analyzed.

### 2.7 Statistical analyses

In Experiment 1, a combined 2 (‘spatial distance’) × 2 (‘digit distance’) repeated measures analysis of variance (rmANOVA) was calculated on the PoR of Tasks 3 to 6, to test for the influence of each factor on hysteresis. All factors were within subject. To resolve a potential interaction, paired, two-tailed *t*-tests (Holm-Bonferroni corrected) were planned as a follow-up.

In Experiment 2, two individual univariate rmANOVAs were calculated: One for Tasks 2 to 4, with three levels of the factor ‘number of drawers’ (9, 7, 5), and one for Tasks 5 and 6, with two levels of the factor ‘skipped drawers’ (with vs. without). All factors were within subject. For the factor ‘number of drawers’, a linear contrast analysis (to test for a linear trend) and paired, two-tailed *t*-tests (Holm-Bonferroni corrected) were planned as follow-ups.

All rmANOVAs were calculated in SPSS (28, IBM Corp., Armonk, NY) to apply the Greenhouse–Geisser correction to the *p*-values of the factor ‘number of drawers’ (if appropriate). Degrees of freedom, however, are reported uncorrected.

## 3 Results

To test if hysteresis was affected by ‘spatial distance’ and/or ‘digit distance’, a 2 × 2 rmANOVA with both factors was calculated on the PoR values of Tasks 3 to 6 from Experiment 1. The interaction of ‘spatial distance’ × ‘digit distance’ was orthogonal and not significant, *F*(1,23) < 1, *p* = 0.980, *η*^2^ < 0.001, indicating that both main effects could be interpreted.

The main effect of ‘spatial distance’ was significant, *F*(1,23) = 10.876, *p* = 0.003, *η*^2^ = 0.094. The PoR was higher for a spatial distance of 1 (13.1%) than for a spatial distance of 2 (7.7%), indicating that participants significantly reduced their percentage of motor plan reuse if the spatial distance between subsequent drawers increased (Figure 3A). The main effect of ‘digit distance’ was not significant, *F*(1,23) < 1, *p* = 0.888, *η*^2^ < 0.001. Participants’ planning behavior was not affected by the digits displayed on the drawers.

To test if hysteresis was affected by ‘number of drawers’, a univariate rmANOVA with three levels (9, 7, 5) was calculated on the PoR values of Tasks 2 to 4 from Experiment 2. The main effect of ‘number of drawers’ was not significant, *F*(2,46) < 1, *p* = 0.693, *η*^2^ = 0.005, indicating that the fraction of motor plan reuse (15.4%) between two subsequent drawers was unaffected by the total number of drawers (Figure 3B).

To test if the presence of task-irrelevant, skipped drawers in the shelf reduced the perceived binding between task-relevant drawers and, thus, the plan reuse, a univariate rmANOVA was calculated on the PoR values of Tasks 5 and 6 from Experiment 2. The main effect of ‘skipped drawers’ was not significant, *F*(1,23) < 1, *p* = 0.490, *η*^2^ = 0.009, indicating that the percentage of motor plan reuse (9.1%) and the perceived binding between task-relevant drawers was unaffected by the task-irrelevant, skipped drawers (Figure 3C).

## 4 Discussion

In a previous study (Schütz and Schack, 2019a), we asked participants to open either every drawer or every second drawer in a vertical shelf in an ordered sequence. The percentage of motor plan reuse (PoR) was considerably smaller in the second condition. In the current study, we asked which of a number of confounded factors that were changed between the two conditions had an influence on the PoR: (1) spatial distance, (2) digit distance, (3) number of drawers, and/or (4) context (presence of task-irrelevant drawers). To this end, participants had to conduct sequential drawer opening tasks. In two experiments, we tested different sets of tasks in which each factor was varied in isolation, to test its influence on the motor hysteresis effect. We expected at least one factor to have a significant influence on the PoR.

Results showed a significant main effect of spatial distance on motor hysteresis: participants reused a smaller percentage of the former motor plan when the spatial distance between subsequent drawers increased. Previous studies have already demonstrated several factors with a similar disruptive effect on motor plan reuse, such as an increase in the mechanical cost of the task (Schütz and Schack, 2013), a decrease in predictability (Schütz and Schack, 2022; Schütz et al., 2011), and an increase in the delay between subsequent trials (Jax and Rosenbaum, 2009). All of these factors were fixed in Experiment 1, and, therefore, could not be responsible for the decrease in PoR. The influence of spatial distance on the PoR found in the current study thus constitutes a novel finding.

Based on the current results, spatial distance can be confirmed as a relevant factor for movement cost optimization in posture planning. This finding, however, cannot be generalized to all domains: Several factors have been shown to influence plan reuse in only one domain. Predictability is a relevant factor in posture planning (Schütz and Schack, 2022) but not in path planning (Jax and Rosenbaum, 2007). A hand switch has a disruptive effect on plan reuse for posture (Schütz and Schack, 2015) but not for path planning (van der Wel et al., 2007). The former plan decays faster in path planning (Jax and Rosenbaum, 2009) than in posture selection (Weigelt et al., 2009) tasks. Thus, future studies should measure persistence in hand path curvature as a function of the angle between subsequent trials in a center-out pointing task, to confirm whether spatial distance is a relevant factor for path planning as well.

From a *cost-optimization* perspective (Schütz et al., 2016), it seems plausible that the PoR is affected by spatial distance: Several studies have demonstrated that posture is a sigmoid function of drawer in a continuous posture selection task (Schütz et al., 2011; Schütz and Schack, 2019a, 2013). As drawer is a proxy for height, this indicates that participants continuously adjust their hand rotation with height. A larger change in spatial distance requires a larger posture adjustment, to ensure that the mechanical cost of opening the next drawer does not increase disproportionately (Schütz et al., 2016). Therefore, the previous motor plan has to be modified to a larger extent to be suitable for the next drawer, which is reflected by a smaller PoR (i.e., a larger percentage of plan modification) in the current study.

Alternatively, one could argue based on the probability that a stored plan can actually be reused for the upcoming drawer: If the probability falls below a certain threshold, a storage of the former plan is no longer cost-efficient. This would also explain why predictability is a relevant factor for plan reuse in a posture selection task: In unpredictable sequences of drawers, the probability that the former plan can be reused is low and, thus, it is more cost-efficient to discard it by default and create a novel plan for each movement. Therefore, hysteresis is absent in randomized sequences of drawers (Schütz and Schack, 2022; Schütz et al., 2011). Similarly, in the current study, the probability that a stored plan can be reused for the next drawer and, consequently, the PoR, decreases with spatial distance.

Critics might point out that the inverse relationship of spatial distance and PoR should be obvious, as a larger change in height has to be reflected in a comparable change of arm posture. Posture in the current study, though, was measured via hand rotation. In Schütz et al. (2017) calculated a principal component analysis on the full arm posture in a sequential drawer opening task and found two groups of coupled joint angles: one that was responsible for height adjustments and one responsible for hand rotation. Only the joint angles responsible for hand rotation were affected by hysteresis. The change in PoR in the current study therefore cannot be explained by a simple change in height, since rotation is regulated independent of drawer height.

The second potential influencing factor, the number of drawers, did not have a significant main effect on hysteresis: the PoR did not change as the number of drawers decreased. In the current study, number of drawers is the only factor that requires global task information. All other factors (spatial distance, digit distance, skipped drawer) can be determined on a local scale, that is, with information about the previous and current step of the sequence alone. Therefore, the finding is in line with the literature, as Schütz and Schack (2019a) showed that a major fraction of the variance associated with plan reuse can be captured by a model that has no (global) information about the number of drawers, but only about the current and the preceding movement.

The lack of a global scope is also evident in binary posture selection tasks: Here, participants switch posture once per sequence and, thus, have the same cognitive cost regardless of the point-of-change. Therefore, the mechanical (and, consequently, the total) cost would be minimal if the point-of-change was located at a height that ensured the most comfortable grasp at each drawer, independent of movement direction. Instead, the point-of-change varies with movement direction (Weigelt et al., 2009; Rosenbaum and Jorgensen, 1992), indicating that the cost criteria are only evaluated on a local scope, that is, from one trial to the next. In contrast, studies on perceptual hysteresis have demonstrated that information of 3-back trials is integrated into the current percept (Fischer and Whitney, 2014) which could, potentially, result in an effect of sequence length on the percept for shorter sequences.

The difference between perception and planning can be explained by the *two visual systems* theory (Goodale and Milner, 1992), which states that separate neural streams process the visual information for perception (ventral stream) and for motor planning (dorsal stream). Despite conflicting evidence (cf. Goodale and Westwood, 2004), a number of studies have found that posture planning in a reaching task is insensitive to various visual illusions, such as the Ebbinghaus illusion (Haffenden and Goodale, 1998; Aglioti et al., 1995), the Ponzo illusion (Brenner and Smeets, 1996; Jackson and Shaw, 2000), or the Müller-Lyer illusion (Westwood et al., 2000). A common finding of these studies was that the perception of a grasped object was affected by the surrounding visual context, whereas grasp posture was not, indicating motor planning was processed by a separate neural stream.

Given this background, it is not surprising that the third potential influencing factor, the context, had no significant effect on hysteresis: the PoR did not vary with the presence/absence of skipped drawers. If context is processed in the ventral visual stream, it simply cannot affect motor planning. In contrast, the last tested influencing factor in the current study, digit distance, is closely linked to the dorsal visual stream (Walsh, 2003). Neurophysiological evidence indicates that numbers are processed in the intraparietal sulcus (IPS; Nieder and Dehaene, 2009; Eger et al., 2003), which is considered not only the locus of an abstract representation of magnitude (Pinel et al., 2004), but also of spatial motor information (Culham and Valyear, 2006). In an fMRI study, Zago et al. (2008) found considerable overlap between number and location processing in the IPS.

Behavioral results support this close link between number and location, but also between number and motor planning: Dehaene et al. (1993) asked participants to indicate the parity status (odd or even) of digits. Results showed that smaller (larger) numbers preferentially elicited a left-hand (right-hand) response, indicating a mental number line that is linked to the left–right coordinates of external space. Lindemann et al. (2007) demonstrated a motor priming effect of digits in a reaching task: low-digit (high-digit) primes preferentially elicited grasps with small (large) aperture, indicating a link between number and motor planning. Despite this, we were unable to demonstrate a significant effect of digit distance on the PoR, that is, on motor planning, in the current study.

A potential explanation for our deviating result could be that participants in the study by Lindemann et al. (2007) did not have direct visual feedback of the grasped object. Cant et al. (2005) showed that such memory-guided grasping can be primed, whereas visually-guided grasping cannot. The authors argued that, for visually-guided movements, a real-time programming of the movement parameters might be more efficient than relying on stored parameters (Cant et al., 2005). While this argument is in line with the finding that the dorsal stream is less affected by memory (Goodale and Westwood, 2004), the same should apply for location, as the loci of number and location overlap (Zago et al., 2008). Yet, we only found a significant effect of spatial distance, but not of digit distance, on motor planning in the current study.

Importantly, a whole range of studies demonstrated a significant influence of memorized posture plans on visually-guided grasping (Rosenbaum and Jorgensen, 1992; Schütz and Schack, 2022; Schütz et al., 2011; Weigelt et al., 2009), including the current one. How can this result be reconciled with a higher efficiency of real-time programming (Cant et al., 2005) and fast memory decay in the dorsal stream (Goodale and Westwood, 2004)? We assume that the central factor for plan reuse still remains predictability: As has been shown by Jax and Rosenbaum (2009), memory traces in the dorsal stream persist sufficiently long to affect motor planning in the subsequent trial, thus reflecting statistical properties of the environment in which we act (Körding and Wolpert, 2006; Trommershäuser et al., 2008, 2006). The high predictability of a sequential task could therefore be reflected by a slower decay of memory traces in the dorsal stream and, thus, result in a stronger influence of the former motor plan.

In the current study, we sought to resolve a confound of four factors that potentially had a disruptive effect on motor plan reuse in a recent study (Schütz and Schack, 2019a), (1) spatial distance, (2) digit distance, (3) number of drawers, and (4) context. When varied in isolation, only spatial distance had a significant influence on motor planning. Future models for posture hysteresis thus should incorporate spatial distance as a factor if distance is varied between tasks. In path planning tasks, an influence of spatial distance on hysteresis still needs to be verified in future studies. Interestingly, the decrease in PoR (due to the increase in spatial distance) in Experiment 1 (5.4%) closely resembled the difference in PoR between the ‘number of drawers’ task (Figure 3B) and the ‘skipped drawers’ task (Figure 3C) in Experiment 2 (6.3%).

This finding likely reflects the difference in spatial distance between both tasks in Experiment 2: the spatial distance was short (1) in Figure 3B, resulting in a larger PoR, and long (2) in Figure 3C, resulting in a smaller PoR. In theory, we have a confound of spatial distance with number of drawers (9/7/5 vs. 5) and digit distance (1 vs. 2) between the two tasks, but since both confounding factors have been shown to have no effect on hysteresis, the difference found in Experiment 2 reaffirms the disruptive effect of an increase in spatial distance on hysteresis for the second participant group. Importantly, the decrease in PoR found in the current study also resembles that of the previous experiment (Schütz and Schack, 2019a), where all four factors were changed simultaneously. This further supports the notion that we were able to isolate the sole driving factor of our four potential candidates: a change in spatial distance between subsequent drawers.

## Data Availability

The datasets presented in this study can be found in online repositories. This data can be found here: doi.org/10.4119/unibi/2988459.
